# Silver nanoparticles synthesis mediated by new isolates of *Bacillus* spp., nanoparticle characterization and their activity against Bean Yellow Mosaic Virus and human pathogens

**DOI:** 10.3389/fmicb.2015.00453

**Published:** 2015-05-13

**Authors:** Essam K. F. Elbeshehy, Ahmed M. Elazzazy, George Aggelis

**Affiliations:** ^1^Department of Biological Sciences, Faculty of Science (North Jeddah), King Abdulaziz UniversityJeddah, Saudi Arabia; ^2^Department of Agricultural Botany, Faculty of Agriculture, Suez Canal UniversityIsmalia, Egypt; ^3^Division of Pharmaceutical and Drug Industries, Department of Chemistry of Natural and Microbial Products, National Research CentreGiza, Egypt; ^4^Unit of Microbiology, Division of Genetics, Cell and Development Biology, Department of Biology, University of PatrasPatras, Greece

**Keywords:** *Bacillus pumilus*, *B. persicus*, *B. licheniformis*, silver nanoparticles, biosynthesis, characterization, bean yellow mosaic virus, human pathogens

## Abstract

Extracellular agents produced by newly isolated bacterial strains were able to catalyze the synthesis of silver nanoparticles (AgNPs). The most effective isolates were identified as *Bacillus pumilus, B. persicus*, and *Bacillus licheniformis* using molecular identification. DLS analysis revealed that the AgNPs synthesized by the above strains were in the size range of 77–92 nm. TEM observations showed that the nanoparticles were coated with a capping agent, which was probably involved in nanoparticle stabilization allowing their perfect dispersion in aqueous solutions. FTIR analyses indicated the presence of proteins in the capping agent of the nanoparticles and suggested that the oxidation of hydroxyl groups of peptide hydrolysates (originated from the growth medium) is coupled to the reduction of silver ions. Energy Dispersive X-ray Spectroscopy confirmed the above results. The nanoparticles, especially those synthesized by *B. licheniformis*, were stable (zeta potential ranged from −16.6 to −21.3 mV) and showed an excellent *in vitro* antimicrobial activity against important human pathogens and a considerable antiviral activity against the Bean Yellow Mosaic Virus. The significance of the particular antiviral activity is highlighted, given the significant yield reduction in fava bean crops resulting from Bean Yellow Mosaic Virus infections, in many African countries.

## Introduction

Silver nanoparticles (AgNPs) attracted the attention of many research groups working on different fields thanks to their unique features and large range of applications (Galdiero et al., 2011), such as in food technology, medicine, agriculture, and environmental technology (Lu et al., 2009; Sastry et al., 2010).

In medicine, AgNPs are considered novel therapeutic agents, displaying important antiviral activity against monkey poxvirus (Rogers et al., 2008), herpes simplex virus (Baram et al., 2010), HIV (Lara et al., 2010), and hepatitis B virus (Lu et al., 2008). The AgNPs synthesized by *Aspergillus* spp. effectively inhibited the proliferation of bacteriophage in host bacteria (Narasimha et al., 2012). Studies on metal nanoparticles, especially on those produced with silver or gold, revealed that nanoparticles exhibit a veridical activity against a broad spectrum of viruses, and surely reduce viral infectivity of cultured cells (Galdiero et al., 2011). Antimicrobial (mainly anti-bacterial), anti-cancerous and anti-inflammatory activities of nanoparticles have been reported (Kim et al., 2007; Kuo et al., 2009). However, despite the financial interest, there are very few reports on the effectiveness of AgNPs against plant viruses. For instance, the effective control of Bean Yellow Mosaic Virus (BYMV), genus Potyvirus family Potyviridae, would be of high interest for many African countries, which can suffer significant yield reductions in fava bean crops upon viral infection leading to considerable economic losses (Radwan et al., 2008).

The antimicrobial activity of AgNPs is attributed to cell death as a result of sequestration and inactivation of vital sub-cellular organelles, for which the silver ions have high affinity (Silver et al., 2006). It has also been suggested that AgNPs inhibit viral nucleic acid replication while their antiviral activity depends on the particle size, as well as on the distribution of interacting ligand/receptor molecules (Lu et al., 2009; Papp et al., 2010).

AgNPs can be produced either by conventional or biological methods. Although the processes that use conventional methods for AgNPs synthesis, i.e., physical, chemical, and hybrid methods S.. (Mazumdar and Ahmed, 2012; Wang et al., 2013) are highly efficient and productive enough, their application in large scale is highly restricted by several factors, including the unsafe chemicals employed, the high demand of energy, the undesirable side-products formed during synthesis and the inefficient so far purification (Kowshik et al., 2003). Moreover, the nanoparticles synthesized by these methods are frequently contaminated with toxic compounds, fact that limits their applicability, especially in medicine (Jain et al., 2011). Alternatively, harmless and satisfactorily effective methods have been proposed for AgNPs nanoparticles synthesis in which selected gram-negative and gram-positive bacteria strains are involved (Vigneshwaran et al., 2007; Prabhu, 2010).

Despite the importance of biosynthesized AgNPs nanoparticles, our understanding of the relevant biochemical pathways is incomplete. Presumably, extracellular molecules of biological origin, such as enzymes, vitamins and polysaccharides may act as reducing and capping agents during nanosilver formation (Collera et al., 2005). It has been suggested that the NADPH-dependent nitrate reductase plays a key role in nanosilver synthesis catalyzing the reduction of silver ion, reaction that induces the nanoparticle formation (Kalimuthu et al., 2008; Kumar et al., 2008).

Several microorganisms are known for their ability to synthesize nanoparticles. Nevertheless, the research for new strains, able to carry out a reliable biosynthesis of nanoparticles with certain properties, such as high stability, monodispersity, or having a particular composition and size, is at the forefront of nanotechnological research. Here, we describe new bacterial strains, able to synthesize AgNPs with important antiviral and antimicrobial activities. The nanoparticles synthesized by three isolates were extensively characterized using physical and chemical methods (including spectrophotometry, electron microscopy, energy dispersive X-ray spectroscopy–EDX and Fourier transform infrared–FTIR analysis). The antimicrobial activity of the nanoparticles was tested against important human pathogens, while their antiviral activity was *in vivo* evaluated against BYMV. In conclusion, the biosynthesized AgNPs are quite stable in aqueous solution showing, especially those synthesized by *Bacillus licheniformis*, an excellent *in vitro* antimicrobial activity against human pathogens and a considerable antiviral activity against BYMV.

## Materials and methods

“The field studies in Jeddah region “King Abdulaziz University, Jeddah 22254 2989, Saudi Arabia Latitude: 21.491089, Longitude: 39.248786” did not involve endangered or protected species; no specific permissions were required for these locations/activities.”

### Isolation of new strains and silver nanoparticles synthesis

The bacterial strains used in this work for AgNPs synthesis were isolated from soil samples (taken from a depth of 5–10 cm) collected from different sites of Jeddah, Saudi Arabia. Pure cultures were established by performing serial dilutions and plating on nutrient agar (NA) (HiMedia, India) medium. Plates were incubated overnight at 28°C. Purified isolates were maintained on NA and refreshed monthly. Based on their ability to rapidly synthesize AgNPs three strains were selected and identified according to the 16S rRNA sequence-based method using the Bacterial 16S rDNA PCR Kit, (Applied Biosystems, USA).

The isolates were grown under aseptic conditions in 25 ml cultures of Nutrient Broth (HiMedia, India), pH 7.0 in 100 ml conical flasks. The flasks were autoclaved (at 121°C for 20 min), inoculated with approximately 10^6^ cells (CFU) and incubated in an orbital shaker (Innova 4230, New Brunswick, NJ, USA), at 30°C under agitation at 220 rpm. After 24 h of incubation the cultures were centrifuged and the supernatants (free of any kind of precipitates) were passed through sterilized membranes of 0.2 μm pore diameter before being used as catalysts for AgNPs synthesis.

The biosynthesis of AgNPs was performed in 100 ml conical flasks containing 20 ml of the supernatant derived from the cultures of the newly isolated strains and silver nitrate at a final concentration of 1 mM. The pH of the reaction mixture was 7.5. The flasks were incubated in an orbital shaker, in dark, at 25°C, under agitation at 120 rpm for 72 h. The progression of the reaction was monitored both visually, as the color of the reaction mixture progressively changed from yellowish to brown, and by a UV-VIS spectrophotometer (see below).

Control flasks containing 20 ml of nutrient broth and silver nitrate at 1 mM were performed in order to confirm that AgNPs synthesis was mediated by extracellular agents of bacterial origin.

### Characterization of silver nanoparticles

The AgNPs samples were subjected to optical absorbance measurements using a UV-Vis spectrophotometer (Labomed UV. 2800 Inc., Culver City, CA, USA) scanning between 200 and 1000 nm at a 1 nm resolution.

The presence of nano-silver elements was confirmed by Energy Dispersive X-ray Spectroscopy (EDX) at 20 keV. Detailed characterization of the size, distribution and morphology of the nanoparticles was performed using transmission electron microscopy (TEM). AgNPs particles were obtained after centrifugation of 20 ml of the reaction mixture at 5000 rpm for 15 min in a JEOL, JEM-200EX (Tokyo, Japan) centrifuge, washed twice with ultra-pure distilled and sterilized water and resuspended in 10 ml sterilized water. One drop of the washed sample was applied to a carbon-coated copper grid and allowed to dry before analysis. Additionally, the Zeta potential and the average size of biosynthesized nanoparticles AgNPs were determined by dynamic light scattering (DLS) using a Malvern Zetasizer Nano ZS (United Kingdom) analyzer at room temperature.

Interactions between proteins present in the supernatants used as source of catalytic agents and the AgNPs were detected using Fourier transform infrared (FTIR) analysis. Specifically, the washed pellets of the AgNPs (obtained as above) were completely dried in a freeze dryer and then analyzed in a FTIR (Perkin-Elmer Spectrum RX1, Shelton, Connecticut) device obtaining the spectrum in the range of 450–4000 cm^−1^ at a resolution of 4 cm^−1^.

### Antimicrobial susceptibility assays

#### Agar well diffusion method

The antimicrobial activity of the AgNPs was *in vitro* tested against human pathogens including *Escherichia coli* ATCC 25922, *Pseudomonas aeruginosa* (clinical isolate), *Shigella sonnei* ATCC 25931, *Klebsiella pneumonia* ATCC 700603, *Staphylococcus epidermidis* ATCC 1228, *Staphylococcus aureus* (methicillin-resistant strain) ATCC 43330, *Streptococcus bovis* ATCC 49147, *Aspergillus flavus* (lab isolate), and *Candida albicans* ATCC 1021 using the agar well diffusion technique. Briefly, 100 μl of cell suspension (5 × 10^6^ CFU) was mixed with 15 ml NA at 45°C in Petri dishes. After cooling, 5 mm diameter wells were opened and 50 μL of 0.1 μg/μL suspended AgNPs in ultra-pure distilled sterile water were aseptically loaded into the wells. Following incubation at 35°C for 18 h, the sensitivity of the organisms was assayed by measuring the diameter of the halo around each well rounding to mm. Control experiments were performed using either the supernatants derived from the bacterial cultures or 1 mM AgNO_3_ solution in NB, instead of AgNPs suspensions.

#### Broth dilution method

The minimal inhibitory concentration (MIC) and the minimal bactericidal concentration (MBC) of AgNPs were determined by the two-fold broth dilution method using the Mueller Hinton II (M-H) broth (Sigma-Aldrich, St. Louis, US) following Clinical and Laboratory Standards Institute (CLSI) guidelines. From a stock suspension of AgNPs (100 μg/ml in ultra-pure distilled water) a two-fold serial dilutions were prepared in the M-H broth. Bacteria cells, found in the exponential growth phase, were inoculated in the wells of a 96-microwell plate containing either M-H broth alone (positive control), or M-H broth containing AgNPs at a final AgNPs concentration ranging from 1.5 to 100 μg/ml. The microplates were then incubated at 37°C for 12 h. The MIC value corresponded to the AgNPs dose that inhibited bacterial growth (when compared to the positive control) and the MBC value, to the AgNPs dose where 100% of the bacterial cells were destroyed. MBC was also evaluated by sub-culturing the content of the first two clear wells obtained in the MIC assay onto M-H agar plates. Bacterial cells viability was measured under a BIOTEK ELx800, Winooski, U.S microplate reader.

#### Bacterial growth kinetics in the presence of AgNPs

The effect of AgNPs on the kinetics of bacterial growth was indicatively studied for *S. aureus* and *E. coli*. Briefly, the M-H broth containing 12.5 μg/ml AgNPs was inoculated with bacterial cells to achieve an initial cell density 1 × 10^8^ CFU/ml. The cultures, performed in 100 ml conical flasks containing 20 ml of M-H broth supplemented with AgNPs, were incubated in a shaker at 37°C and under agitation at 100 rpm for 24 h. The growth curves were drawn by using the values of optical density (OD_600_) obtained during growth.

#### Virus

The anti-viral activity of the AgNPs was tested *in vivo* against the BYMV, which is a positive-sense, single-stranded RNA virus. BYMV was collected from fava bean plants displaying severe yellow mosaic, mottling, crinkling, size reduction and leaf distortion and stored in 70% ethanol for later identification. The samples were serologically examined for BYMV, using specific polyclonal antibodies with direct ELISA test. Specifically, all samples were examined in triplicate using conventional DAS-ELISA according to the manufacturer's instructions (Sanofi-Santi animal, France), reading the optical density at λ = 405 nm in an ELISA micro-well reader (Dynatech Immunoassay MR 7000) (Clark and Adams, 1977). Leaf samples that gave positive reaction with specific BYMV antibodies were used for BYMV inoculation of fava bean cv. Giza 3. In particular, the virus inoculum was prepared after grinding the infected leaves in a mortar containing 50 mM potassium phosphate buffer pH 7.0, at a ratio1:2 w/v. The homogenate was filtrated through a double layer of muslin, and the leaves of healthy fava bean seedlings were dusted with carborundum and rubbed gently with a cotton swab previously dipped into the virus suspension containing 10^−3^–10^−4^ virions. The fava bean seedlings were preserved in a controlled insect proof screen house at 24 −27°C for at least 2 weeks after inoculation. Besides to macroscopic examinations for symptoms, the ELISA test was used as a presence test of the virus as described in Radwan et al. (2008).

##### RT-PCR for BYMV identification

Total RNA was extracted and purified with the SV total RNA isolation system (Promega) with the spin protocol recommended by the manufacturer. 50 mg of BYMV-infected leaves from broad bean leaves were ground in liquid nitrogen using a mortar and pestle and stored at −20°C until extraction (Duraisamy et al., 2011).

Following total RNA extraction, the QIAGEN Single Step RT-PCR Kit (Qiagene, Hilden, Germany) was used for virus identification. To amplify the BYMV genome, primers were designed and evaluated from the coat protein region by reverse transcription-polymerase chain reaction (RT-PCR). The forward 5′CAGTTTATTATGCAGCGG3′ and reverse 5′GTTATCATCAATCTTCCTGC3′ primers were used according to Hiroyuki and Tsuda (2005). The RT-PCR products were separated on 1% agarose gels in 0.5X TBE buffer, stained with the GelStar nucleic acid gel stain (Lonza, USA) and visualized by UV illumination using Gel Documentation System (Gel Doc 2000, Bio-Rad, USA). Fragments were sized using a 100 bp marker.

##### Treatment of infected plants by silver nanoparticles

Seeds of *Vicia faba*, cv. Giza 3 were planted in plastic pots of 100 cm^3^ with sterilized soil and grown under usual and propitious conditions for bean plant. The humidity was maintained at around 70%. After 21 days of growth, plants of similar size were chosen and separated into three Groups (i.e., I, II, III), each one of which consisting of eight Subgroups (of three replicates each). Subgroup [1] included healthy fava bean seedlings (negative control); Subgroup [2]–[4] included healthy faba bean seedlings treated (sprayed) with AgNPs synthesized by the three isolates; Subgroup [5] included fava bean seedlings inoculated with BYMV (positive control) and Subgroup [6]–[8] included fava bean seedlings inoculated with BYMV and treated with AgNPs synthesized by the three isolates. The identity of the groups is as follows: Group I–plants treated with AgNPs simultaneously with BYMV infection; Group II–plants treated with AgNPs before virus infection by 72 h; Group III–plants treated with AgNPs 24 h after virus infection. The plant viral infection was diagnosed by visual inspection of the affected leaves.

Almost all parts of the leaves were sprayed with 0.1 μg/μl AgNPs. 21 days after inoculation the disease severity (DS%) was recorded according to the following formula (Yang et al., 1996):

(1) $$\begin{array}{l} \text{DS }(\%)\\ =\frac{\left(\text{disease grade }\times \text{ number of plants in each grade}\right)}{\left(\text{total number of plants}\times \text{ highest disease grade}\right)}\times \;100 \end{array}$$

The scale used for the disease grade was: 0 = no symptoms; 1 = light crinkling and mottling; 2 = mild crinkling and mosaic; 3 = severe crinkling, mosaic and size reduction; and 4 = deformation.

##### Electron microscopy of Vicia faba tissues

For thin sectioning, tissue pieces from veins and mesophyll of the discolored areas of young leaves were processed according to Radwan et al. (2008). Briefly, tissues were fixed in 4% glutaraldehyde solution in 0.05 M phosphate buffer (pH 6.8) for 2 h, post-fixed in 1% osmium tetroxide for 2 h, stained overnight in 2% aqueous uranyl acetate, dehydrated in ethanol, and embedded in Spurr's medium. Thin sections were stained with lead citrate and observed in a JOEL-JEA100 CX electron microscope unit. Controls consisted of leaf tissues from a PCR-negative seedling.

#### Statistical analysis

All data were expressed as the mean of three independent experiments. Data were analyzed using One-Way analysis of variance (ANOVA) followed by least significant difference (LSD) test for comparisons of all treatments at 5% probability level using CoSTAT software program (C0Hort Computer Software, Berkeley, CA, USA).

## Results

### Strains' identification

**Figure 1:**
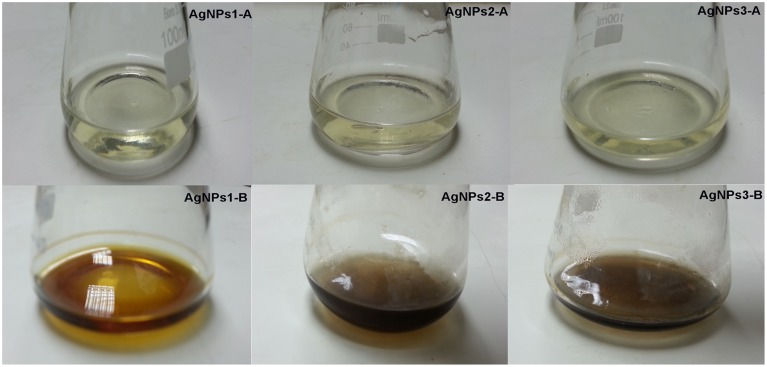
**Discoloration of bacterial culture supernatant due to AgNO_3_ reduction**. Reduction of silver ions by *Bacillus pumilus, B. persicus*, and *B. licheniformis* extracellular agents, as evidenced by color change of culture supernatant from yellow to dark brown. The control flasks **(A)** contain cell-free filtrates without silver nitrate and flasks **(B)** contain cell-free filtrates with silver nitrate. The yellowish color of the reaction mixtures turned to brown within 24 h of incubation in dark.

**Figure 2:**
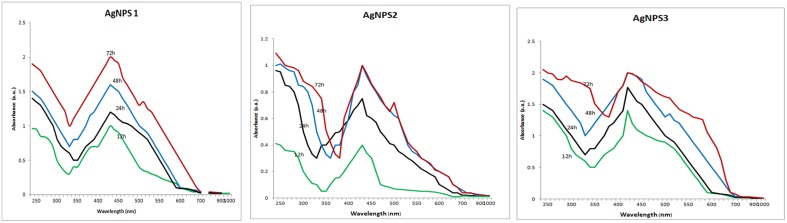
**UV–VIS spectra of the biosynthesized silver nanoparticles**. UV–VIS spectra of silver nanoparticles synthesized by extracellular agents of *Bacillus pumilus, B. persicus*, and *B. licheniformis*, were recorded as a function of the reaction time. The peak at 425 nm corresponds to the surface plasmon resonance of silver nanoparticles.

The screen of isolates for AgNPs rapidly synthesizing strains resulted in the initially named strains NPs-1, 2, and 3. The particular strains were found to produce extracellular agents that efficiently catalyze the synthesis of AgNPs, as it was suggested by the rapid and stable color change of the AgNO_3_ solution from yellow to dark brown within a few hours of exposure (Figure 1). Indeed, the dark brown color remained stable for at least 48 h, indicating the long-term stability of the synthesized nanoparticles, an important property when biotechnological applications are considered. The AgNPs formation was confirmed by the presence of characteristic surface plasmon resonance (SPR) of silver nanoparticles at 450 nm measured by UV-Visible spectrophotometer (Figure 2).

No AgNPs synthesis was observed in control flasks containing 20 ml of NB (instead of bacterial supernatant) and AgNO_3_ at 1mM, confirming that AgNPs synthesis was mediated by extracellular agents of bacterial origin.

The above strains were identified to species-level using PCR amplification of the 16S rRNA gene, BLAST analysis, and comparison with known sequences of the GenBank nucleotide database as *Bacillus pumilus, B. persicus*, and *B. licheniformis* and the respective nanoparticles were named NPs-1, NPs-2, and NPs-3. The sequences of *B. pumilus, B. persicus*, and *B. licheniformis* were deposited at the Genbank and received the accession numbers of KJ743246, KJ743245, and KJ743244, respectively. Detailed information concerning the 16S rRNA of the three isolates is provided in the Figures S1–S3 and Table S1 (see Supplemental Materials).

### Synthesis and characterization of silver nanoparticles

The above isolates were cultivated on NB medium for 24 h. Subsequently, the supernatants (free of any kind of precipitates) were used as catalysts for AgNPs synthesis. Silver nitrate exposure to the cell-free supernatants of *B. pumilus, B. persicus*, and *B. licheniformis* resulted in a time-dependent color change of the reaction mixture from yellowish to brown (Figure 1), suggesting AgNPs biosynthesis. Further, the formation of AgNPs was detected and monitored over time by UV–Vis absorption spectrum scanning in the range of 200–1000 nm (Figure 2). As illustrated in the UV-VIS spectra the absorbance intensity gradually increased with time without any shift of the wavelength in which the maximum absorbance was observed, denoting a continuous reduction of silver nitrate and, consequently, an increase in AgNPs concentration.

**Figure 3:**
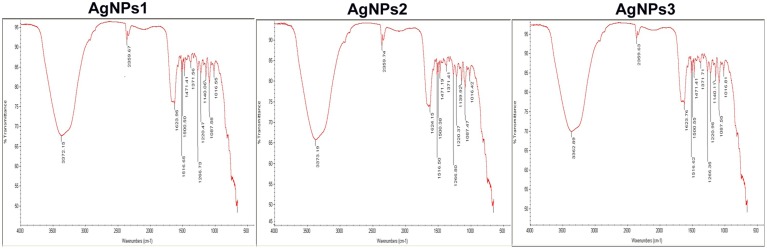
**FTIR spectra of the biosynthesized silver nanoparticles**. FTIR spectrum of silver nanoparticles synthesized by extracellular agents of *Bacillus pumilus, B. persicus*, and *B. licheniformis*, after 48 h of incubation. The bands seen at 3372, 2359, and 1650–1000 cm^−1^ correspond to **(A)**: the stretching vibrations of primary amines, **(B)**: C = O extend vibrations of carboxylic acids, aldehydes, and ketones, and **(C)**: methylene scissoring vibrations, respectively.

Comparison of FTIR spectra of NPs-1, NPs-2, and NPs-3 indicated only minor differences in both the bands position and the absorption intensity (Figure 3). Specifically, the analysis revealed the existence of a band at 3373 cm^−1^ in the FTIR spectrograms and a strong peak at 3300–3500 cm^−1^. The peak at about 2359 cm^−1^ was also remarkable. Peaks were also observed at 1650 cm^−1^ and at 1600–1000 cm^−1^.

**Figure 4:**
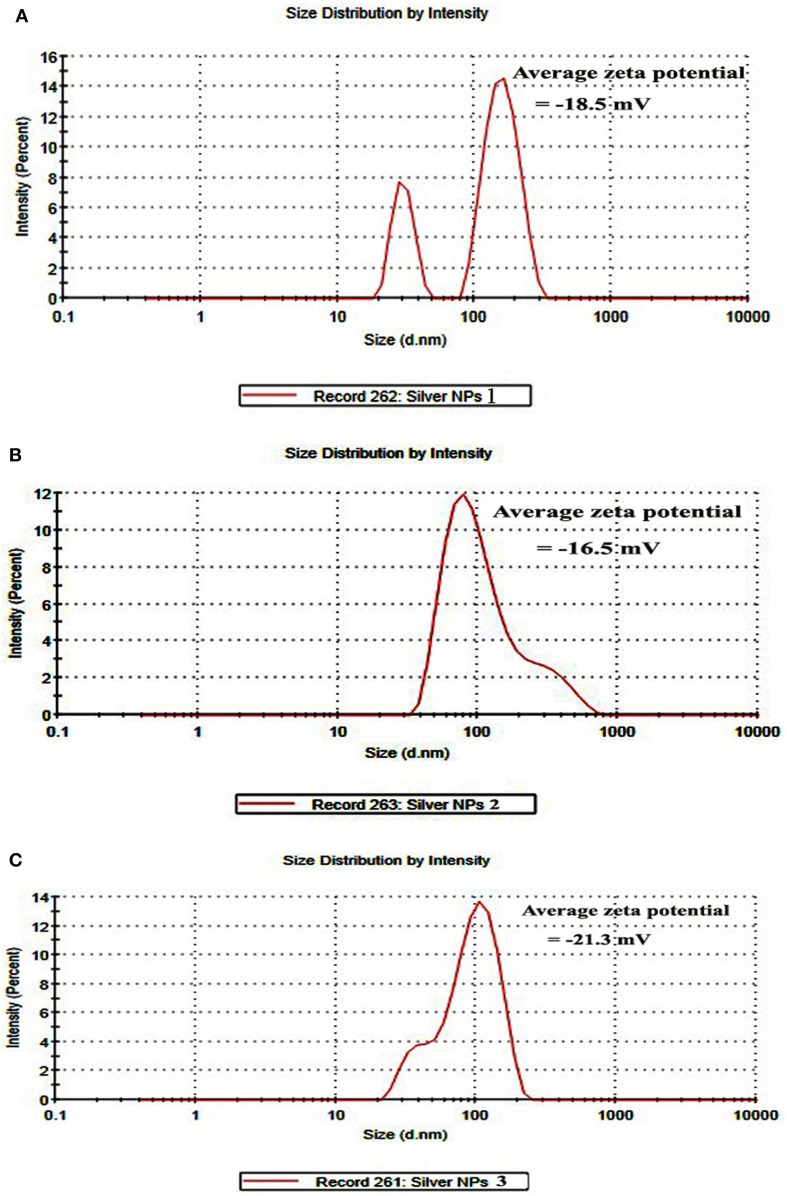
**Size distribution intensity graph of NPs-1 (A), NPs-2 (B), and NPs-3 (C) as revealed by DLS**.

DLS and Zeta potential have been used to determine the size of the particles and their potential stability in the colloidal suspension, respectively. Figures 4A–C show the size distribution of AgNPs synthesized by *B. pumilus, B. persicus, and B. licheniformis* which was found to be in average 80, 92, and 77 nm, respectively. Furthermore, the particles carried a charge of −18.5, −16.6, and −21.3 mV, for *B. pumilus, B. persicus*, and *B. licheniformis*, respectively. Therefore, all silver nanoparticles synthesized by the three *Bacillus* strains showed a negative zeta potential and were stable at room temperature.

**Figure 5:**
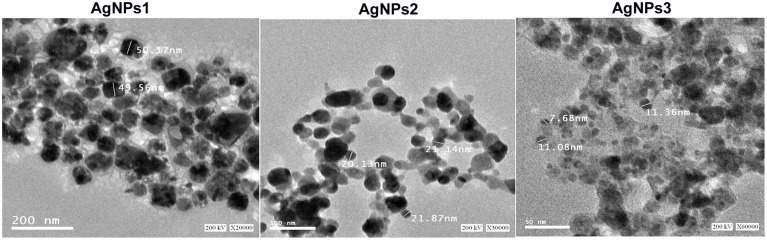
**TEM images of the biosynthesized silver nanoparticles**. A representative TEM image recorded from the drop coated film of the silver nanoparticles synthesized by extracellular agents of *Bacillus pumilus, B. persicus*, and *B. licheniformis* and estimation of nanoparticles diameter.

The TEM images confirmed the production of AgNPs at nano-scale, most of them being mono-dispersed with triangular, hexagonal and spherical shapes (Figure 5). The nanoparticles, surrounded with an organic layer, were not agglomerated even within the aggregates.

**Figure 6:**
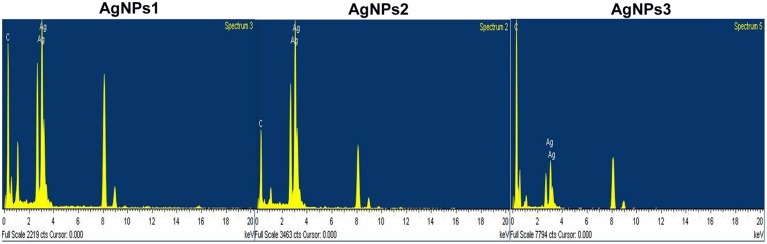
**EDX spectra of the biosynthesized silver nanoparticles**. EDX spectra recorded from a film of silver nanoparticles synthesized by extracellular agents of *Bacillus pumilus, B. persicus*, and *B. licheniformis* with different X-ray emission peaks labeled.

The EDX analysis of the AgNPs proved the existence of an elemental silver signal (Figure 6). Other EDX signals emitted from O, N, and C atoms were also noticed.

### Antimicrobial activity of silver nanoparticles

Interestingly, the biosynthesized AgNPs showed significant antimicrobial activity against several human pathogenic bacteria (both Gram positive and negative) and fungi (Table 1). Control wells containing the supernatants derived from the bacterial cultures showed no inhibition (Figure S4). Although control containing AgNO_3_ in NB showed an antimicrobial activity (data not shown), this activity increased considerably when AgNPs were used. NP-3 synthesized by *B. licheniformis* exhibited the highest inhibitory effect against Gram negative bacteria including *E. coli, K. pneumoniae, S. sonnei, P. aeruginosa*, followed by Gram positive *S. epidermidis*, MRSA and *S. bovis*. Their antifungal activity against *A. flavus* and *C. albicans* was also significant.

**Table 1:** **Inhibition zone (mm) caused by silver nanoparticles synthesized by extracellular agents of *Bacillus pumilus* (NPs-1), *B. persicus* (NPs-2), and *B. licheniformis* (NPs-3) on cultures of human pathogens bacteria and fungi**.

	**NPs-2**	**NPs-1**	**NPs-3**
*Escherichia coli*	25 ± 2	15 ± 2	15 ± 1
*Shigella sonnei*	22 ± 2	18 ± 2	15 ± 0
*Pseudomonas aeruginosa*	20 ± 0	18 ± 1	16 ± 0
*Klebsiella pneumonia*	23 ± 1	18 ± 1	15 ± 0
*Streptococcus bovis*	14 ± 0	13 ± 0	12 ± 0
*Staphylococcus epidermidis*	18 ± 2	10 ± 0	6 ± 1
*Staphylococcus aureus*	15 ± 1	12 ± 0	16 ± 2
*Aspergillus flavus*	15 ± 0	10 ± 0	10 ± 1
*Candida albicans*	20 ± 2	20 ± 0	13 ± 1

*For details see text. The values are the means of three experiments ±SD*.

Data concerning MIC and MBC of biosynthesized NPs-1,-2, and-3 against the tested organisms are presented in Table 2. These results indicate that NPs-3 had the lowest MICs and MBCs values when compared to those of NPs-1 and NPs-2. This fact can be attributed to the size of NPs-3 that is the smaller one of all AgNPs tested in this study. The highest values of MIC and MBC were recorded using NPs-2 against the Gram positive bacteria *S. bovis* and *S. aureus*, while the respective lowest values were observed for *K. pneumonia, S. sonnei*, and *E. coli* when NPs-3 were used as antimicrobial agent. Undoubtedly, Gram negative bacteria, having a thinner cell wall than the Gram positive, were more susceptible to the bactericidal activity of AgNPs.

**Table 2:** **Determination of Minimum Inhibition Concentration (MIC) and Minimum Bactericidal Concentration (MBC) of silver nanoparticles synthesized by extracellular agents of *Bacillus pumilus* (NPs-1), *B. persicus* (NPs-2), and *B. licheniformis* (NPs-3)**.

	**NPs-1**		**NPs-2**		**NPs-3**	
	**MIC (μg/ml)**	**MBC (μg/ml)**	**MIC (μg/ml)**	**MBC (μg/ml)**	**MIC (μg/ml)**	**MBC (μg/ml)**
*Escherichia coli*	12.5±0.3	25.0±0.0	12.5±1.0	25.0±0.5	3.2±0.0	12.5±0.7
*Shigella sonnei*	6.3±1.1	12.5±0.4	12.5±1.0	12.5±0.6	3.2±0.0	3.2±1.0
*Pseudomonas aeruginosa*	25.0±0.0	25.0±0.0	12.5±0.0	25.0±1.0	6.3±1.3	12.5±0.3
*Klebsiella pneumonia*	12.5±0.0	25.0±0.6	12.5±0.0	12.5±1.7	3.2±0.0	3.2±0.0
*Streptococcus bovis*	50.0±0.0	<100	100.0±0.0	100.0±0.6	25.0±2.0	50.0±0.0
*Staphylococcus aureus*	12.5±0.0	12.5±0.0	12.5±1.3	50.0±1.0	12.5±0.0	12.5±1.7

*For details see text. The values are the means of three experiments +SD*.

**Figure 7:**
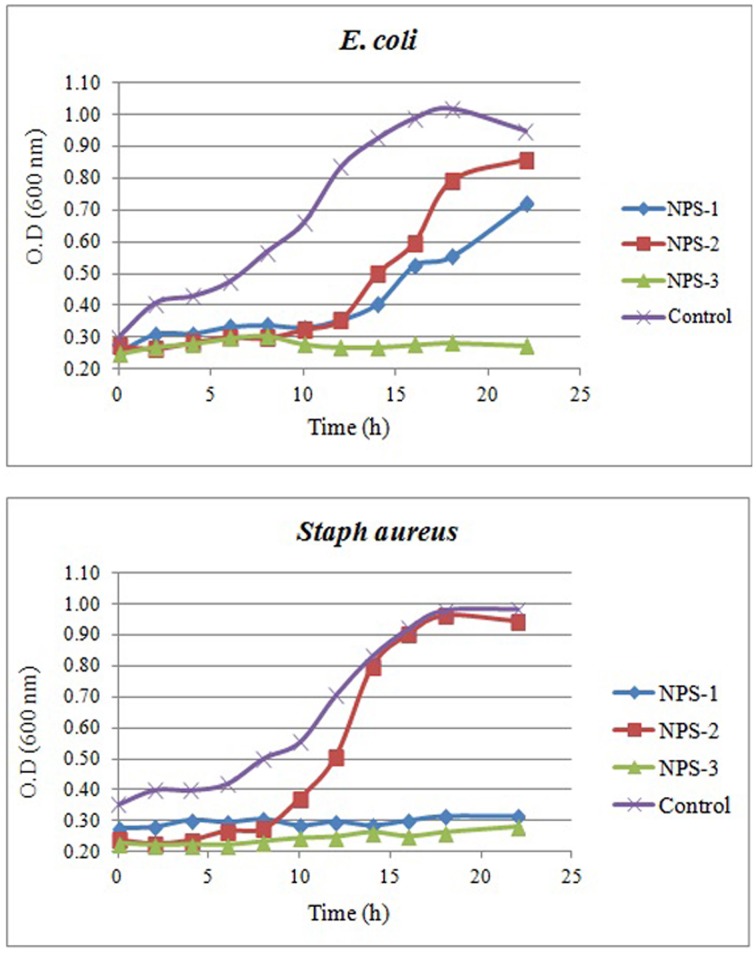
**Growth curves of *E. coli* (A) and *Staph. aureus* (B) in the presence of AgNPs (NPs-1, 2, and 3) and comparison to the growth curves obtained in the absence of AgNPs (control)**.

The growth curves of *E. coli* and *S. aureus* in the presence AgNPs are shown in Figures 7A,B. Interestingly, the presence of NPs-3 into M-H broth provoked a complete inhibition of both *E. coli* and *S. aureus*, while after 8 h almost all treated cells were destroyed. However, the cells treated with NPs-2, were slightly or not at all inhibited. NPs-1 prolonged the lag phase of *E. coli* for 10 h and completely arrested the growth of *S. aureus*.

### Antiviral activity of silver nanoparticles against BYMV

**Figure 8:**
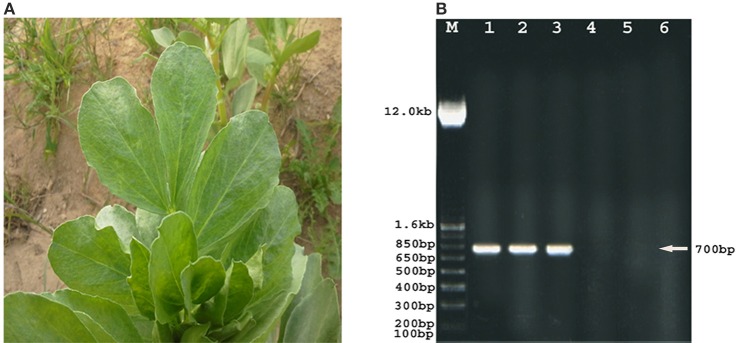
**Phenotypic and molecular evidence of the presence of Bean Yellow Mosaic Virus (BYMV) on fava bean plants**. Symptoms caused by natural BYMV infection on fava bean leaves showing severe mosaic, crinkling, and size reduction **(A)**. Agarose gel electrophoresis analysis of amplified BYMV-cp gene fragment (lanes 1–6) **(B)**. RT-PCR products of six BYMV samples showing amplified BYMV-cp, gene fragment of the correct size 700 bp (arrow) in lanes 1, 2, and 3. BYMV-cp, gene is absent in lanes 4, 5, and 6 (healthy plants). M: DNA ladder marker.

**Figure 9:**
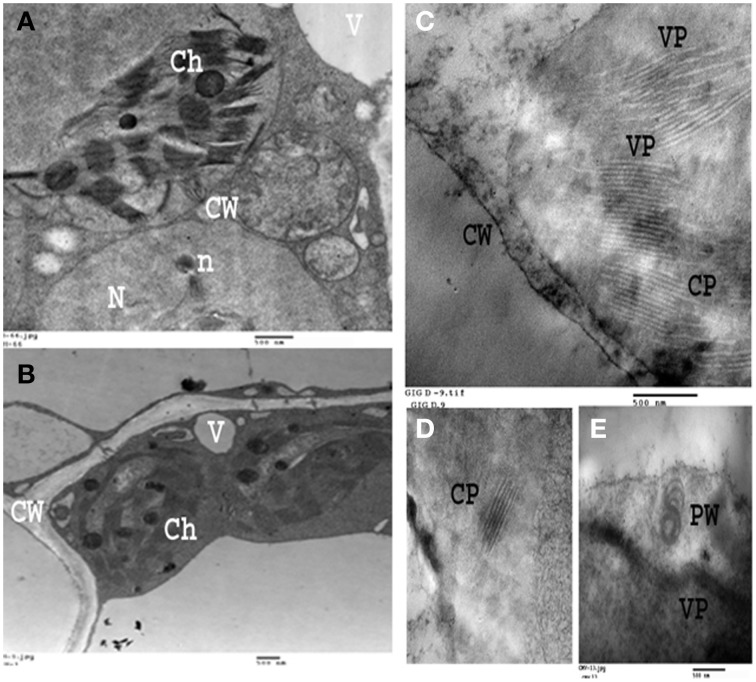
**Electron micrograph of an ultra-thin section of healthy and infected broad bean leaves. (A,B)**: Ultra-thin section of healthy broad bean leaves. **(C–E)**: Ultrathin section of light green area in broad bean leaves infected with BYMV showing laminated aggregate of Crystalline particles (CP), Pinwheel inclusions (PW), Viral particles (VP). Cell organelles (i.e., Ch, chloroplasts; V, vesicles; CW, cell wall; N, nucleus) are also displayed.

Leaves of fava bean infected with BYMV showed severe symptoms, including yellow mosaic, mottling, crinkling, size reduction and deformation (Figure 8A), symptoms that were absent from the non-infected leaves. Moreover, BYMV coat protein (cp) gene was detected using RT-PCR (Figure 8B) in six samples of infected leaves, whereas no product was amplified from healthy plants. In ultrathin sections of broad bean leaves inoculated with BYMV, viral particles elongated and flexuous, 750 nm long and 15 nm wide, as well as inclusion bodies containing elongated pinwheel structures, typical of potyviruses, were observed (Figure 9).

**Figure 10:**
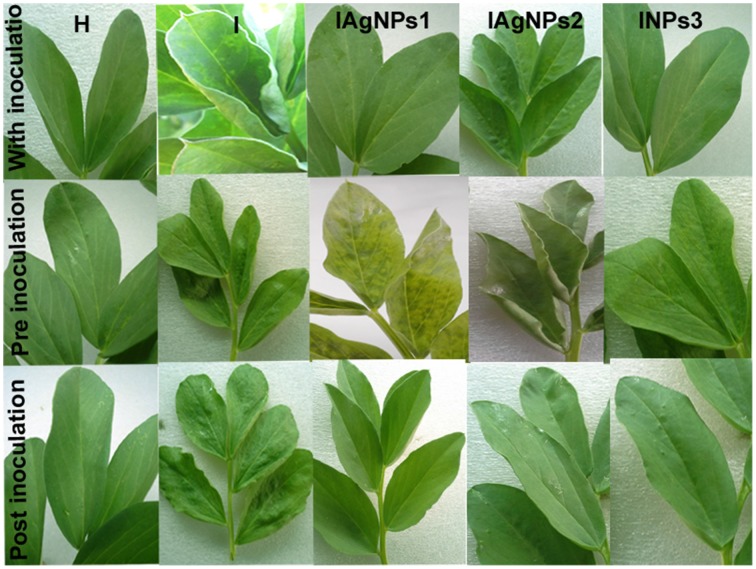
**Effect of silver nanoparticles synthesized by extracellular agents of *Bacillus pumilus, B. persicus*, and *B. licheniformis* on BYMV infected *Vicia faba L*. cv. *Giza3* and non-infected plants**. The nanoparticles were sprayed simultaneously with virus inoculation (experimental Group I); before inoculation (Group II) or after inoculation (Group III). Systemic severe mosaic, crinkling, and size reduction were observed in the case of infected leaves. These symptoms were reduced or completely disappeared when plants were treated with silver nanoparticles synthesized by the isolates, especially those of *B. licheniformis* of Group III. For details see text.

AgNPs synthesized by *B. pumilus, B. persicus*, and *B. licheniformis* had no effect on the healthy plants (data not shown), which was a prerequisite for the potential use of nanoparticles in agriculture. However, the treatment of infected plants with AgNPs induced different plant responses, depending on the treatment time (pre-, along with- or post-viral infection). In particular, the pre-infection treatment with AgNPs (i.e., 72 h prior to inoculation), had no effect neither on the virus concentration nor on the severity of the disease (Figure 10, Table 3). On the contrary, remarkable results were observed in the post-infection treatment (i.e., 24 h after inoculation) in which every one of NPs-1, NPs-2, and NPs-3 prevented all destructive symptoms caused by the virus, while weak BYMV symptoms were observed when plants were treated by AgNPs simultaneously with the infection.

**Table 3:** **Effect of silver nanoparticles synthesized by extracellular agents of *Bacillus pumilus, B. persicus*, and *B. licheniformis* on virus concentration, percentage of infection and disease severity (DS) of fava bean, cv. Giza3 infected by Bean yellow mosaic virus under greenhouse conditions**.

**Treatment**	**Virus concentration by DAS-ELIZA Group**			**Infection (%) Group**			**DS (%) Group**		
	**I**	**II**	**III**	**I**	**II**	**III**	**I**	**II**	**III**
No treatment	1.744	1.431	1.150	100	100	100	100	100	100
NPs-1	0.411	1.199	0.095	0	100	0	0	75	0
NPs-2	0.532	1.288	0.415	89	100	34	44	100	8
NPs-3	0.400	1.112	0.091	0	100	0	0	50	0

*Group I: plants treated with silver nanoparticles simultaneously with BYMV infection; Group II: plants treated with silver nanoparticles before virus infection; Group III: plants treated with silver nanoparticles after virus infection. For details see text. The values are means of three determinations; In Elisa test the positive (infected leaves with symptoms) and the negative (infected leaves without symptoms) controls are 1.492 and 0.113, respectively*.

Quantitative data on the effect of AgNPs on BYMV concentration, percentage of infection and disease severity are summarized in Table 3. Symptoms were relieved by all nanoparticle treatments performed 24 h after inoculation, particularly by the treatment with NPs-3, which led to an important decrease in virus concentration, percentage of infection and disease severity. Moderate reduction in all symptoms was exhibited when the nanoparticles sprayed in parallel with the infection, while weak or zero reduction in virus concentration, percentage of infection and disease severity was observed when AgNPs were sprayed at the pre-viral infection stage.

## Discussion

Biologically synthesized AgNPs are promising therapeutic agents demonstrating significant antimicrobial and antiviral activities (Rogers et al., 2008; Kuo et al., 2009; Galdiero et al., 2011). Although a number of biosynthesized nanoparticles have been described and characterized for their ability to inhibit/destroy microbial cells and viruses, the investigation for new nanoparticles with specific physicochemical and biological properties remains at the forefront of nanotechnological research. The ability of three newly isolated *Bacillus* strains, belonging to the species *B. pumilus, B. persicus*, and *B. licheniformis*, to perform AgNPs synthesis is reported in this paper. The biosynthesis was mediated by extracellular agents and the synthesized AgNPs proved to be effective against important human pathogens, bacteria and fungi, and BYMV.

The formation of AgNPs occurred after exposing silver nitrate to the cell-free supernatants of the bacterial cultures and the progression of the nanoparticle synthesis was monitored using UV-VIS spectrophotometry. AgNPs have an absorption peak at 425 nm, attributed to their SPR property, probably due to the stimulation of longitudinal plasmon vibrations (Kumar and Mamidyala, 2012). SPR property is also responsible for the color change of the reaction mixture from yellowish to brown (Chaudhari et al., 2012; Yamal et al., 2013). Therefore, the increase of the absorbance at 425 nm is a reliable criterion indicating AgNPs synthesis (Thu et al., 2013). Although *B. pumilus* extracellular material gradually synthesized AgNPs throughout the period 12–72 h, in the case of *B. persicus* and *B. licheniformis* there were no changes of the absorbance intensity after 48 h, indicating the completion of the reaction, as well as a good stability of the biosynthesized NPs-2 and NPs-3. Nevertheless, the maximum values of absorbance vary according to the size and shape of AgNPs, in agreement with earlier reports (Kumar and Mamidyala, 2012; Mittal et al., 2014).

With the intention to determine the functional group responsible for Ag^+^ ions reduction, as well as the capping agent of the reduced AgNPs, FTIR analyses were carried out. Specifically, FTIR analysis can be used for the detection of potential interactions between silver salt and extracellular proteins that are involved in AgNPs formation (Saha et al., 2010), while it has been suggested that this analysis provides information about the binding of proteins to AgNPs leading to nanoparticles stabilization (Jain et al., 2011). For these reasons FTIR has been routinely used by several researchers in nanoparticles characterization. Similar FTIR spectra were obtained by NPs-1, NPs-2, and NPs-3, all of them indicating the existence of proteins in the capping agent of the nanoparticles, and also that the secondary structure of proteins has not been affected as a consequence of the reaction with silver ions or the binding to AgNPs. This conclusion is supported by the band at 3373 cm^−1^ in the FTIR spectrogram, which is specific to the frequency of extending vibration of primary amines, and the strong band peak at 3300–3500 cm^−1^, which is characteristic of N-H stretching vibrations, indicating strong hydrogen bonding (Saha et al., 2010). The appearance of a band at about 2359 cm^−1^, which is assigned to C = O extend vibrations of carboxylic acids, aldehydes and ketones, was remarkable, indicating that the oxidation of the hydroxyl groups of hydrolysates (originated from the medium peptides) are coupled to the reduction of silver ions. The bands observed at 1650 cm^−1^ is a definite indicator of linkages between the amides I and II (Sharma et al., 2012). The following vibrations observed at 1600–1000 cm^−1^ may be indicative of methylene scissoring vibrations from the proteins in the bacterial filtrate (Sharma et al., 2012).

According to Meléndrez et al. (2010) zeta potential analysis can be used to gain further insights concerning the stability of AgNPs. The values of zeta potential for the AgNPs produced in this study indicate a long-term stability of the colloids, which could be attributed to the presence of microbial proteins that cover nanoparticles stabilizing them. AgNPs obtained using *B. licheniformis* gave the highest negative value of zeta potential (i.e., −21.3 mV), which indicates a good stability.

DLS analysis revealed that the size of AgNPs synthesized by *B. pumilus, B. persicus, and B. licheniformis* was in average 80, 92, and 77 nm, respectively. The morphology of the AgNPs has been illustrated using TEM that provides descriptive images revealing also the AgNPs distribution (Nagati et al., 2012). It is important that the AgNPs are not agglomerated, even within the aggregates, and coated with an organic layer, indicating stabilization of the nanoparticles by a capping agent. Capping agents are key-components of nanoparticles allowing a perfect dispersion of nanoparticles in the bio-reduced aqueous solution (Kathiresan et al., 2010). We obtained highly confirmative results by EDX analysis, proving the existence of elemental silver. The optical absorption peak of silver nano-crystals arises at about 3 KeV, which is similar to that of metallic silver nano-crystals due to SPR (Park et al., 2007). Other EDX signals emitted from O, N, and C are probably due to X-ray emission from proteins presented in the cell free filtrates (Das et al., 2010) that can bind to nanoparticles either through free amino groups or cysteine residues (Mandal et al., 2005). These results are closely correlated with the TEM results.

The antibacterial activity of AgNPs increased considerably with the decrease of the particles size. Therefore, the smallest AgNPs (having an average diameter = 77 nm) synthesized by *B. licheniformis* exhibited the higher inhibitory effect mainly against Gram negative, but also against Gram positive bacteria. Their antifungal activity was also significant. Similar conclusions are derived when MIC and MBC values are considered. Furthermore, the bacterial growth curves obtained in the presence of AgNPs indicated a significant prolongation of the lag phase provoked by AgNPs, while NPs-3 completely inhibited growth of *E. coli* and *S. aureus*.

Accordingly, it has been reported that antimicrobial activity is dose and size dependent and more obvious against Gram negative than Gram positive bacteria (Singh et al., 2008). Presumably, the Gram negative cell wall, composed of thin peptidoglycan layer, is more susceptible to AgNPs permeation when compared to the Gram positive cell wall, which is consisted of a thicker peptidoglycan layer, creating an effective barrier against the AgNPs penetration, assumption that is in agreement with previous reports (Rai et al., 2009). Marambio-Jones and Hoek (2010) suggest that Ag^+^ ions of AgNPs attached to the negatively charged cell surface alter the physical and chemical properties of cell membranes and disturb important functions such as permeability, osmoregulation, electron transport and respiration. According to Rajeshkumar and Malarkodi (2014) the silver nanoparticles may also directly interact with the microbial cells. Specifically, silver ions can inhibit the respiratory chain enzymes and the permeability to phosphate and protons (e.g., by interrupting transmembrane electron transfer, oxidizing cell components, disrupting and penetrating the cell covering making it susceptible to the reactive oxygen species (ROS) or dissolving heavy metal ions that cause damages). Moreover, further damage to bacterial cells can be caused by permeating the cell, where they interact with proteins, DNA and other sulfur- and phosphorus-containing cell constituents (Nayak et al., 2015).

BYMV infection caused severe symptoms to fava bean plants such as yellow mosaic, mottling and so on (see above). Similar symptoms have been recorded in previous papers (Arneodo et al., 2005; Radwan et al., 2008; EL-Bramawy and El-Beshehy, 2012) for other beans (i.e., *Phaseolus vulgaris* and *Glycine max*) infected with BYMV or related potyviruses such as Zucchini yellow mosaic virus. Further, the presence of BYMV in infected leaves was confirmed using RT-PCR, while viral particles, as well inclusion bodies containing typical of potyviruses elongated pinwheel structures, were observed. The development of pinwheel structures associated with laminated aggregates composed of closely apprised proteinaceous sheets is a characteristic feature of Potyviruses belonging to subdivision II (Radwan et al., 2008).

The most effective treatment of infected plants with AgNPs was that applied 24 h after inoculation (post-infection treatment). In particular, the post-infection treatment with NPs-3 led to an important decrease in virus concentration, percentage of infection and disease severity. Correspondingly, it has been reported that the AgNPs are effective against a prototype arena virus when administered early after initial virus exposure (Speshock et al., 2010). These findings may suggest that the AgNPs activity is more dramatic at the early phases of viral replication. However, weak or zero reduction in virus concentration, percentage of infection and disease severity was observed when AgNPs were sprayed at the pre-viral infection stage, indicating inability of the AgNPs to activate the inducible systemic resistance of the plant against BYMV infection.

Remarkably, the most effective AgNPs (causing nearly complete suppression of viral infection) were those produced by *B. licheniformis* having the smallest size (9–11 nm), findings which are in accordance with those reported in Lu et al. (2008). Additionally, in the later paper the authors, analyzing monodispersed AgNPs for their ability to inhibit replication of hepatitis B virus (HBV), reported that the large AgNPs were toxic for the cell as well, but the smaller particles showed a minor toxicity at the concentration needed for inhibition of HBV replication. The same trend, with a more pronounced activity, was observed in influenza virus inhibition assays demonstrating that the activity clearly depends on the particle dimension and the spatial distribution of the interacting ligand/receptor molecules (Papp et al., 2010). It has been shown that, besides the immediate interaction with glycoprotein of the virus surface, AgNPs may enter the cell and fulfill their antiviral activity through interactions with the viral nucleic acids (Galdiero et al., 2011).

In conclusion, this research paper provides new insights in the synthesis of AgNPs through reductive reactions that are induced by biological agents of bacterial origin. Newly isolated strains of *B. pumilus, B. persicus*, and *B. licheniformis* produced extracellular agents (i.e., peptides) that were able to catalyze the synthesis of AgNPs. Specifically, the oxidation of the hydroxyl groups of extracellular peptides was probably involved in the reduction of silver ions leading to nanoparticles formation. The synthesized AgNPs were quite stable in the reaction mixture, mono-dispersed, of various shapes and were coated with an organic layer in which proteins participated. The nanoparticles, especially those synthesized by *B. licheniformis*, showed an excellent *in vitro* antimicrobial activity against important human pathogens and a considerable antiviral activity against BYMV infection, which is of importance for many African countries, since it may cause a significant yield reduction in fava bean crops. This information is valuable for future registration and labeling of the AgNPs as antiviral agents for crop protection and for further elucidation of the mechanisms involved in virus inactivation.

## Author contributions

Conceive and designed the experiments: EE, AE, GA; Performed the experiments: EE, AE; Analyzed the data: EE, AE, GA; Wrote the paper: AE, GA.

### Conflict of interest statement

The authors declare that the research was conducted in the absence of any commercial or financial relationships that could be construed as a potential conflict of interest.
