# Supplementary material for: Silver nanoparticles synthesis mediated by new isolates of Bacillus spp., nanoparticle characterization and their activity against Bean Yellow Mosaic Virus and human pathogens
Source: Front Microbiol. 2015 May 13;6:453. doi: 10.3389/fmicb.2015.00453 (PMC4429621; doi:10.3389/fmicb.2015.00453)
Supplement: Supplementary file 1 [file Presentation1.PDF]

Figure S-1. Molecular Phylogenetic analysis of Strain NPs-1isolate by Maximum Likelihood method.

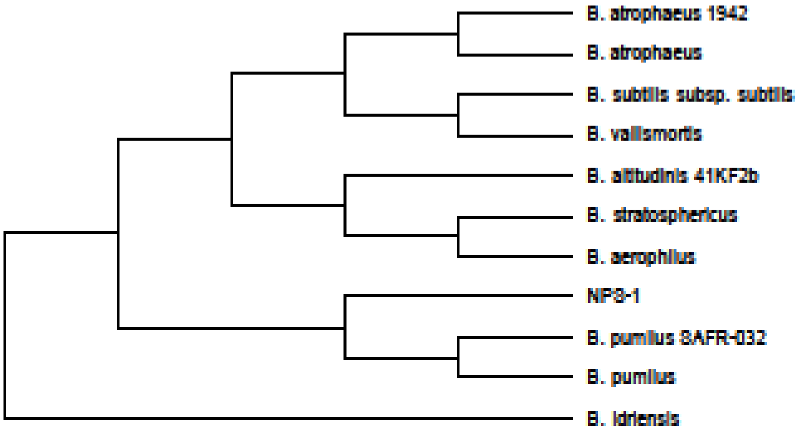

Figure S-2. Molecular Phylogenetic analysis of Strain NPs-2isolate by Maximum Likelihood method.

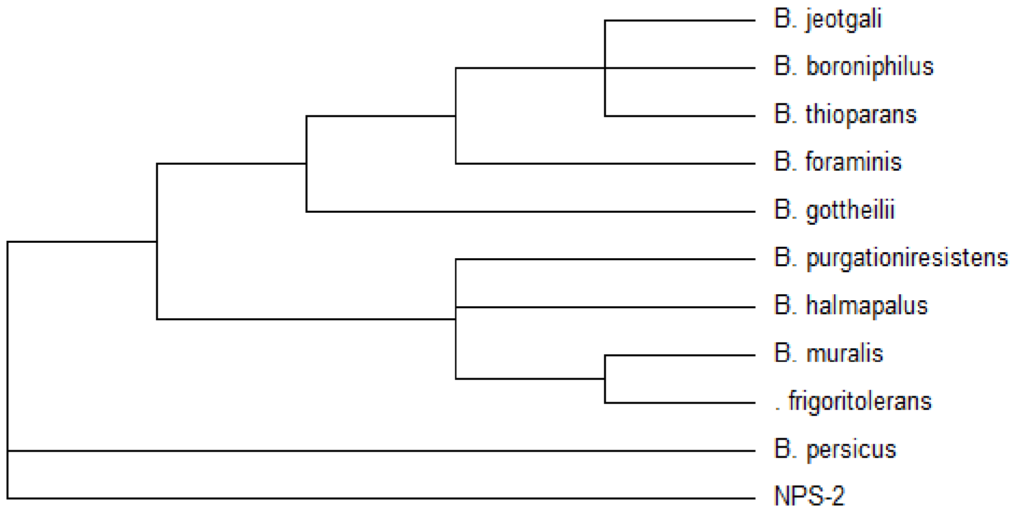

Figure S-3. Molecular Phylogenetic analysis of Strain NPs-3isolate by Maximum Likelihood method.

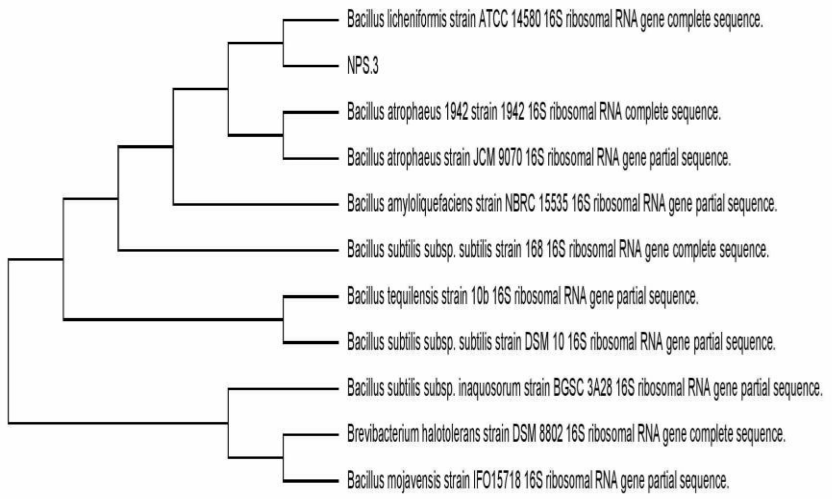

The evolutionary history was inferred by using the Maximum Likelihood method based on the Tamura-Nei model [1]. The bootstrap consensus tree inferred from 450 replicates [2] is taken to represent the evolutionary history of the taxa analyzed [2]. There were a total of 1038 positions in the final dataset. Evolutionary analyses were conducted in MEGA6 [3].

1. Tamura K. and Nei M. (1993). Estimation of the number of nucleotide substitutions in the control region of mitochondrial DNA in humans and chimpanzees. Molecular Biology and Evolution 10:512-526.

2. Felsenstein J. (1985). Confidence limits on phylogenies: An approach using the bootstrap. Evolution 39:783-791.

3. Tamura K., Stecher G., Peterson D., Filipski A., and Kumar S. (2013). MEGA6: Molecular Evolutionary Genetics Analysis version 6.0. Molecular Biology and Evolution 30: 2725-2729.

Figure S4

Antimicrobial activity of AgNps-1, 2 and 3 against *Candida albicans* and *E. coli,* control wells containing the supernatants derived from the bacterial cultures showed no inhibition.

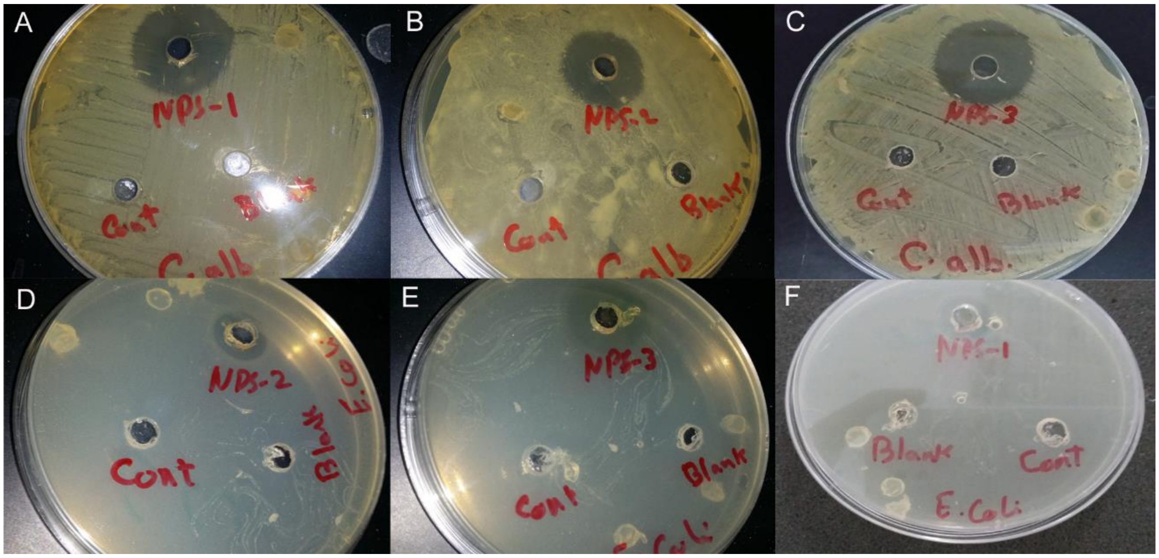

Table S1. Sequence analysis of the three isolates using NCBI BLAST analysis tool showing the maximum sequences similarity values of 98 - 99% to known NCBI database strains.

Isolate that synthesizes	Description	E value	Ident.
NPs-1	*Bacillus pumilus*	00	98%
NPs-2	*Bacillus persicus*	00	99%
NPs-3	*Bacillus licheniformis*	00	99%
